# Elective caesarean section without routine indwelling bladder catheterization – impact on mobilization and patient satisfaction

**DOI:** 10.1186/s12884-026-09763-1

**Published:** 2026-08-08

**Authors:** Anni Ilonen, Antti Väänänen, Antti Puhakka, Hanna Vihervaara, Marja Kaijomaa

**Affiliations:** 1 Department of obstetrics and gynaecology, University of Helsinki and Helsinki University Hospital, Helsinki, Finland; 2 Department of Anaesthesiology and Intensive care, University of Helsinki, Helsinki University Hospital, Helsinki, Finland; 3 University of Eastern Finland, Kuopio, Finland; 4 Department of Obstetrics and gynaecology, HUCH/Women’s hospital, Haartmaninkatu 2, PO BOX 140, HUS, FI-00029 Finland

**Keywords:** cesarean section, early mobilization, enhanced recovery after surgery, urinary catheterization, patient satisfaction

## Abstract

**Background:**

Global caesarean section rates have continuously increased. Therefore, a remarkable number of women could benefit from Enhanced Recovery After Surgery (ERAS^®^) protocol aiming to reduce perioperative stress and achieve early recovery. In 2019 the ERAS^®^ protocol for caesarean section was initiated in the Helsinki University Women’s Hospital and the Espoo Delivery Hospital in Finland. Among other improvements, the routine use of indwelling urinary catheter was discontinued among healthy women with no significant preoperative risk factors.

**Methods:**

This retrospective case-control study was carried out at the Espoo Hospital in Finland. Data were collected retrospectively from the hospital’s electronic patient information system. The study included 208 women undergoing full term elective caesarean section. Group 1 (*n* = 104) routinely had an indwelling urinary catheter placed: half of them had no pre-specified time for catheter removal (*n* = 52) and half of them had an indwelling urinary catheter placed for 8 to 12 h (*n* = 52). Group 2 (*n* = 104) had a perioperatively placed urinary catheter only if needed. The primary outcome was the time from the end of the operation to patient mobilization, while secondary outcomes were the length of hospital stay and overall patient satisfaction.

**Results:**

Group 2 without a routine indwelling urinary catheter had a significantly shorter median time to mobilization (median = 5.8 h) than group 1 (median = 9.1 h) *p* < 0.001. In the multivariable analysis, the absence of the urinary catheter was the strongest factor associated with earlier mobilization. There was no difference in length of hospital stay, patient satisfaction or intraoperative blood loss between the groups and no bladder injuries were recorded.

**Conclusion:**

Patients without a routine indwelling urinary catheter mobilized significantly faster compared to patients with a routinely placed urinary catheter. We found no difference in length of stay or patient satisfaction between the groups. These results support limiting the routine use of indwelling urinary catheter among healthy women undergoing caesarean section.

## Background

Global caesarean section rates, defined as the proportion of all births delivered by cesarean section, have continuously increased and remain on an upward trend [1]. In 2024, the caesarean section rate in Finland was 20.3%, representing the highest rate ever recorded [2].

Enhanced Recovery After Surgery (ERAS^®^) protocol aims to reduce perioperative stress and achieve early recovery for patients. The protocol was first introduced in 1997 by Danish professor Henrik Kehlet [3] and over the years this protocol has spread from colorectal surgery to numerous major surgical specialties [4]. The utilization of ERAS^®^ protocol has resulted in a significant reduction in postoperative complications, length of hospital stay and total treatment costs [5–7].

In 2018–2019, the ERAS^®^ society published guidelines for perioperative management of caesarean delivery. These guidelines give recommendations for limiting preoperative fasting, multimodal pain management, fluid management, prevention of maternal hypothermia, early mobilization, immediate removal of urinary catheter, and prevention of nausea [8–10]. There are differences in clinical protocols between hospitals, but in most protocols, attention is paid to minimal fasting time, early mobilization, and shorter duration of urinary catheterization [11]. According to the ERAS^®^ society’s recommendation, the urinary catheter should be immediately removed after caesarean section if inserted preoperatively [10]. A routine use of an indwelling urinary catheter has been shown to be unnecessary [12] and without catheterization the time to ambulation and hospital discharge is shorter [13]. Catheter-associated urinary tract infections (CAUTI), defined as a symptomatic urinary tract infection where an indwelling urinary catheter was in place minimum two consecutive days on the date of event or the day before, are common healthcare-associated infections. Most important risk factor for CAUTI is prolonged use of urinary catheter, as the daily risk of bacteriuria with an indwelling urinary catheter is 3% to 7% [14]. However, there are varying opinions on both the necessity and appropriate duration of urinary catheterization [15].

In 2019, the ERAS^®^ protocol was initiated at the Helsinki University Women’s Hospital and subsequently at the Espoo Delivery Hospital. A recent study by Vihervaara et al. [16] showed that in Espoo Delivery Hospital pre-specified time for removal of urinary catheter after 8, 10, or 12 h based on randomization, was associated with significantly faster mobilization compared to routine protocol of urinary catheterization. Subsequently the routine use of indwelling urinary catheter was discontinued among healthy women with no significant preoperative risk factors.

The objective of this study was to evaluate the outcomes of the elective caesarean section without routine indwelling urinary catheter. The primary outcome was the time from the end of the operation to patient mobilization, while secondary outcomes were the length of hospital stay and overall patient satisfaction.

## Methods

This retrospective case-control study was carried out at the Espoo Delivery Hospital, which is affiliated with the Helsinki University Women’s Hospital, in Finland. The Espoo Hospital is the second largest delivery hospital in Finland handling over 4 000 deliveries annually [2]. It manages low-risk caesarean sections, therefore ruling out patients with placenta accreta spectrum, severe pre-eclampsia, type I or II diabetes, coagulopathy, antenatal anticoagulation treatment or severe obesity (BMI over 45 kg/m^2^). Thus, the study population comprised a homogeneous population of low risk parturients.

Data were collected retrospectively from the hospital’s electronic patient information system (Apotti/Epic) based on midwives’ patient documentation and medical record. The primary outcome was the time to first mobilization (standing or walking), measured from the end of the procedure and based on standardized midwife observations documented in the patient’s medical records. Secondary outcomes were the length of hospital stay, calculated from the beginning of surgery to discharge, and overall patient satisfaction. The birthing experience was routinely inquired at the postpartum ward by the midwives before discharge i.e. 24–48 h after surgery and patient-reported satisfaction was measured by a Visual Analogue Scale (VAS) ranging from 0 to 100 mm (0 = complete dissatisfaction; 100 = highest possible satisfaction).

We compared two groups of women undergoing full-term elective caesarean section. In total, there were 208 participants, 104 in each group. Women in group 1 routinely had an indwelling urinary catheter placed: half of them had no pre-specified time for catheter removal (*n* = 52) and half of them had an indwelling urinary catheter placed for 8 to 12 h (*n* = 52). Women in group 1 comprised enrolled participants in a pilot study and their controls. The recruitment period for group 1 was from December 2022 to December 2023, and the data were originally collected for a study concerning shorter catheter treatment. For this study, Vihervaara et al. [16] calculated that to detect a three-hour difference in the mobilization time, the needed sample size was 44 (α = 0.05, β = 0.20). To account for potential missing data, the sample size was set at 52 participants per group. Group 2 consisted of a consecutive cohort of women undergoing an elective caesarean section. Women in group 2 were operated without a routine indwelling urinary catheter and perioperative catheterization was performed only if needed. Women in group 2 were preoperatively given 0.4 mg of tamsulosin and were instructed to void just before being transferred to the operating theatre. Recruitment for group 2 was done when the protocol for cesarean section without indwelling urinary catheter was successfully adopted, from October to December 2024. Accordingly, 104 consecutive participants in chronological order were recruited for group 2.

The caesarean sections were all performed under spinal anesthesia (bupivacaine 10–12 mg, fentanyl 15 µg, morphine 120 µg), mainly by junior doctors in the early stages of their careers. Hypotension was prevented by routine phenylephrine infusion, initiated at the time of spinal anesthesia administration. All parturients undergoing elective caesarean delivery under spinal anesthesia have received the infusion according to an institutional protocol in place since 2013. The postoperative pain medication routine was to administrate ibuprofen 600 mg and paracetamol 1 g three times daily and, if necessary, additional doses of oxycodone 5–10 mg. In accordance with the hospital policy, no opioids were prescribed at the time of discharge from the hospital.

In group 2, the bladder was drained empty by a single use catheter at the end of the caesarean section. In the postnatal ward the women were instructed to void regularly, and the emptying of the bladder was routinely verified by an ultrasound bladder scan. In accordance with the hospital protocol, intermittent catheterization was performed if voiding was unsuccessful after two attempts, or if the estimated residual urine volume by bladder scan was over 500 ml. If the residual urine volume was over 1 000 ml, an indwelling catheter was inserted for a period of 24 h.

Statistical analyses were performed using SPSS Statistics for Windows 28.0.0 (IBM Corp., Armonk, N.Y., USA). Pearson’s chi-square test was used for categorical variables. T-test and Mann-Whitney U test were used for continuous variables after normality tests. A multiple linear regression analysis was conducted to examine the relationship between time to mobilization and eight potential cofactors. The level of statistical significance was set at *p* < 0.05.

## Results

The characteristics of two groups are shown in Table 1. There was no significant difference in mean maternal age between the groups (34.0 vs. 34.3, *p* = 0.64). Body mass index in group 2 was higher than in group 1, but the difference was not statistically significant (29.0 vs. 30.1, *p* = 0.06). No differences were observed between the groups in either the duration of surgery (61 vs. 58, *p* = 0.11) or intraoperative blood loss (650 vs. 500, *p* = 0.39). No bladder injuries were recorded.

**Table 1:** Characteristics of two groups undergoing elective caesarean section

	Group 1 routine catheter treatment *n* = 104	Group 2 catheter only if needed *n* = 104	
Age, mean (SD), years	34.0 (5.0)	34.3 (4.6)	*p* = 0.64 ^1^
BMI (kg/m^2^), median (IQR)	29.0 (6.4)	30.1 (5.4)	*p* = 0.06 ^2^
Diagnoses (%)			
fear of childbirth	42.3	56.7	*p* = 0.04 ^3^
breech presentation	28.8	23.1	*p* = 0.34 ^3^
previous uterine scar	20.2	18.3	*p* = 0.73 ^3^
Duration of operation, median (IQR), minutes	61 (31)	58 (22)	*p* = 0.11 ^2^
Intraoperative blood loss, median (IQR), milliliters	650 (400)	500 (390)	*p* = 0.39 ^2^
Perioperative catheterization (%)	100	25	
Time to mobilization, median (IQR), hours	9.1 (5.8)	5.8 (2.6)	*p* < 0.001 ^2^
Time to discharge from hospital, median (IQR), days	2.2 (1.0)	2.2 (0.7)	*p* = 0.24 ^2^
Patient satisfaction VAS from 0 to 100, median (IQR)	90 (12)	95 (12)	*p* = 0.73 ^2^

^1^ t-test, ^2^ Mann-Whitney U test, ^3^ Chi-Square test

*Abbreviations*: *BMI* body mass index, *IQR* interquartile range, *VAS* visual analogue scale

**Fig. 1:**
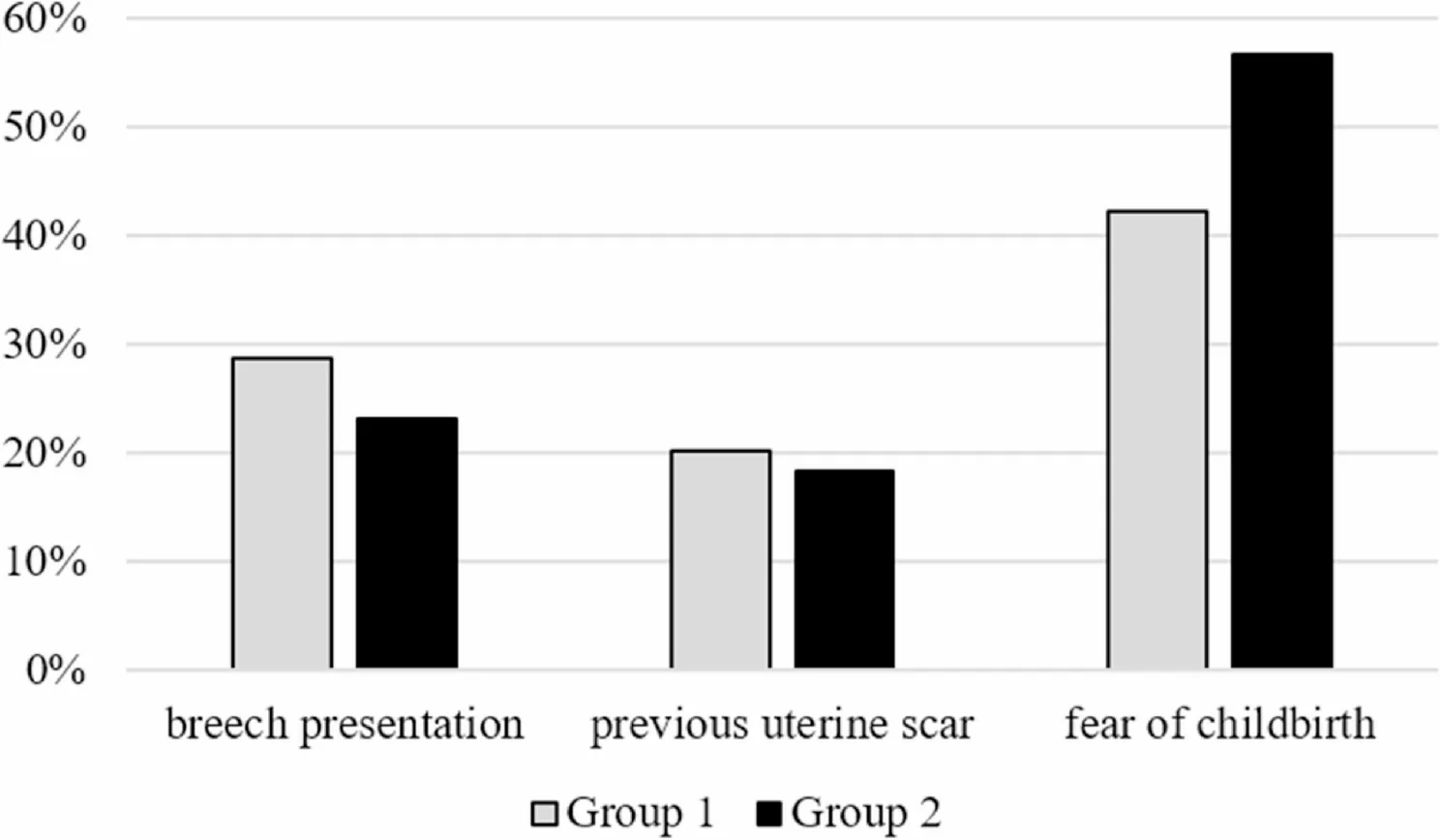
The diagnoses across the groups undergoing caesarean section. The most common diagnosis in both groups was fear of childbirth, followed by breech presentation and maternal care due to uterine scar from previous surgery. There were significantly more diagnoses of fear of childbirth in group 2, *p* = 0.04

The indications for elective cesarean section are shown in Fig. 1. Fear of childbirth was the most common indication for elective cesarean section in both groups, with a significantly higher incidence in group 2 (42.3% vs. 56.7%, *p* = 0.04). The next most common indications were breech presentation (28.8 vs. 23.1, *p* = 0.34) and maternal care for a uterine scar from previous surgery (20.2 vs. 18.3, *p* = 0.73), with no significant differences in their incidence between the groups. In addition, there were singular cases of gestational diabetes, fetal macrosomia, disproportion, and other abnormalities of pelvic organs in both groups.

**Fig. 2:**
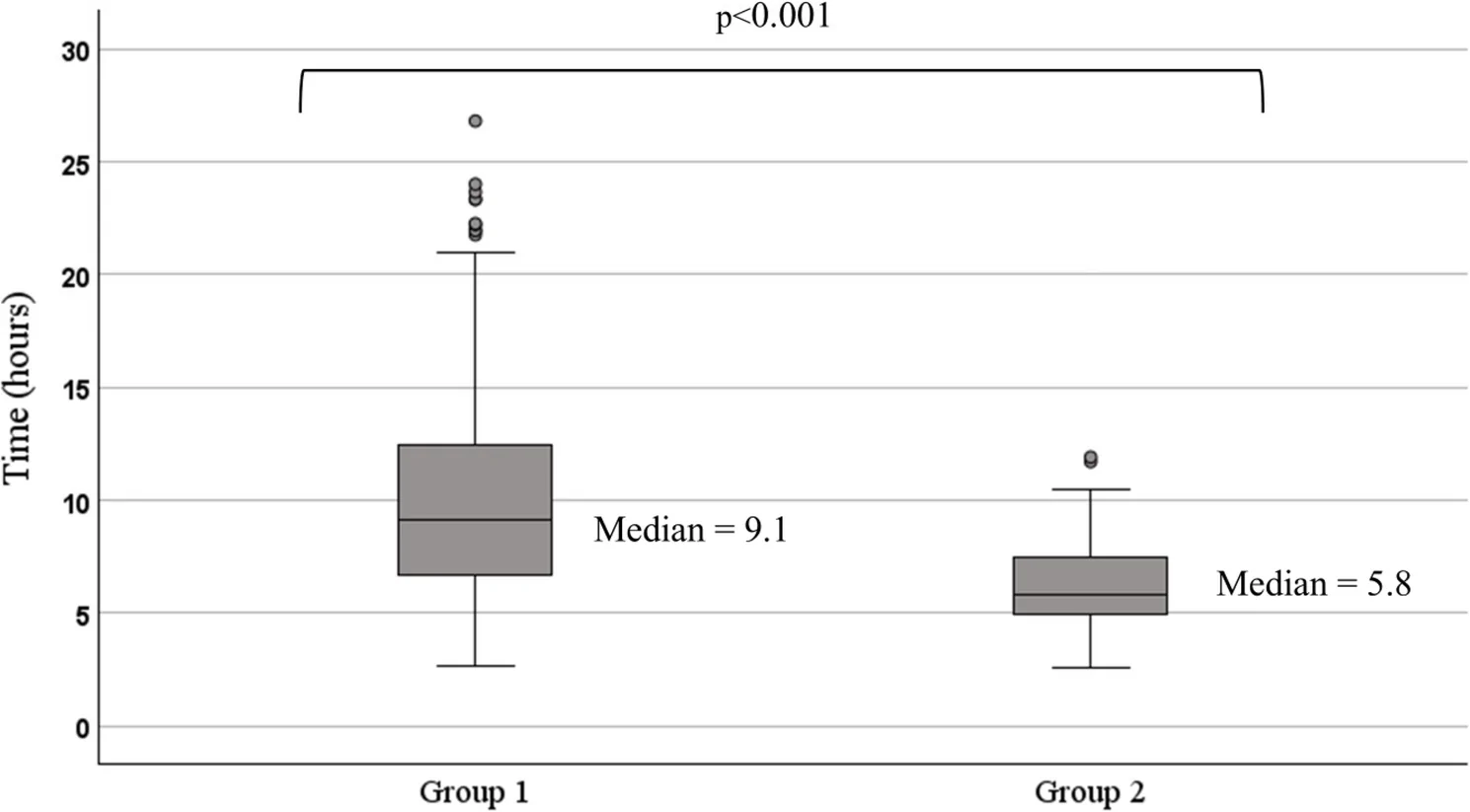
Time to the first mobilization after the caesarean section. Group 2 had a significantly shorter median time to patient mobilization, *p* < 0.001

**Fig. 3:**
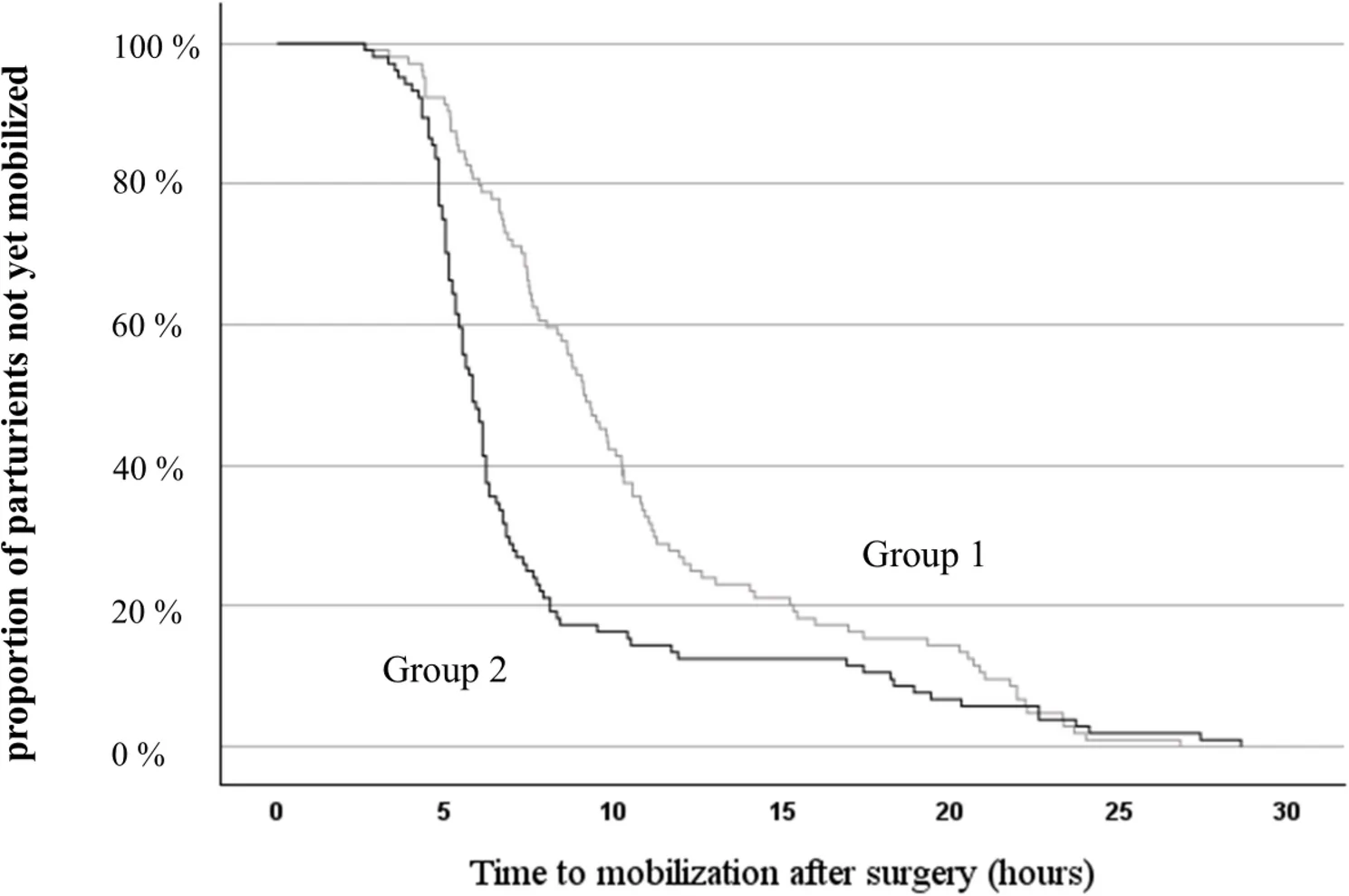
Kaplan-Mayer survival curve: time to mobilization after the caesarean section. Group 2 (dark grey) represents a real-world cohort of consecutive parturients that were intended to be operated without the indwelling urinary catheter. Group 1 (light grey) was operated with routine catheter treatment. Group 2 mobilized significantly faster compared to group 1

The time to patient mobilization in group 2 (median = 5.8 h) was significantly shorter than in group 1 (median = 9.1 h) *p* < 0.001, as demonstrated in Figs. 2 and 3. No significant differences were detected in the length of hospital stay (2.2 vs. 2.2, *p* = 0.24) or in patient satisfaction (90 vs. 95, *p* = 0.73).

The median duration of catheterization in group 1 was 13.4 h (IQR 11.5). In group 2, a total of 26 patients were catheterized perioperatively due to instructions from obstetricians or anesthesiologists. The reasons for perioperative catheterization were abdominal adhesions, greater than average blood loss, excessive fluid treatment or an additional dose of anesthetics. Among the 78 patients without perioperative catheterization, the median time from the end of caesarean section procedure to the first spontaneous voiding was 7.6 h (IQR 7.0). In group 2, a total of 16 patients (21%) got a second 0.4 mg dose of tamsulosin, and 37 patients (47%) needed at least one (mean = 1.1) postoperative catheterization in the postnatal ward, including 11 patients who got an indwelling urinary catheter. The mean duration of catheterization with an indwelling catheter was 22.1 h (SD 6.5). The reasons for the catheterization were unsuccessful voiding (73%), feeling unwell (24%), and abnormal bleeding (3%). No patient was discharged home with a catheter. Patient satisfaction among re-catheterized patients (median VAS 95) did not differ from other patients.

As shown in Table 2, a multiple linear regression analysis was conducted to examine the relationship between time to mobilization and eight predictors: age, body mass index, diagnosis of breech presentation, fear of childbirth and previous uterine scar, duration of operation, intraoperative bleeding and perioperative indwelling catheterization. The overall model was statistically significant, F (8,199) = 7.81, *p* < 0.001, and explained 21% of the variance in time to mobilization (R^2^_adj_ = 0.21). Diagnoses of breech presentation and fear of childbirth had small to moderate association with shorter time to mobilization, whereas the absence of perioperative indwelling catheterization was moderately associated with a significantly shorter time to mobilization.

**Table 2:** Regression coefficients for predicting time to mobilization after caesarean section (in hours)

Variable	B	95% Cl	β	t	*p*
Age	0.003	[-0.15, 0.16]	0.003	0.04	0.97
Body mass index	0.05	[-0.11, 0.21]	0.04	0.57	0.57
Breech presentation	-2.76	[-5.22, -0.29]	-0.21	-2.20	0.03
Previous uterine scar	0.15	[-2.22, 2.53]	0.10	0.13	0.90
Fear of childbirth	-2.82	[-4.99, -0.65]	-0.24	-2.56	0.01
Duration of operation	-0.02	[-0.06, 0.02]	-0.06	-0.93	0.35
Intraoperative bleeding	0.001	[-0.002, 0.003]	0.03	0.47	0.64
Perioperative indwelling catheterization	4.92	[3.36, 6.47]	0.41	6.23	< 0.001

R^2^_adj_ = 0.21 (*N* = 208, *p* < 0.001). *Cl* confidence interval for B

## Discussion

The objective of this study was to evaluate the impact of changes in the urinary catheterization protocol on postoperative mobilization and patient satisfaction. In the multivariable analysis, the absence of the urinary catheter was the strongest factor associated with earlier mobilization. The change in clinical practice did not affect patient satisfaction or length of hospital stay. No bladder injuries were recorded.

We have not noticed any increase in the need for phenylephrine dosing after the implementation of tamsulosin premedication. Although the exact efficacy of tamsulosin in the prevention of post-operative urinary dysfunction has not been firmly established, there are studies describing its use also in women following pelvic surgery [17] and studies reporting reduced postoperative urinary retention without significant adverse effects [18].

Use of intrathecal morphine as part of the multi-modal elective cesarean analgesia is acknowledged to represent the golden standard of post-cesarean delivery analgesia regime. One of the often-stated side effects of neuraxial opioids, such as morphine, is the negative impact on the bladder function [19]. It is noteworthy that even with the currently used relatively high dose (120 µg) over half of the parturients in our cohort without urinary catheter did not require re-catheterization at the postnatal ward. The optimal dose of intrathecal morphine has been subject to debate, and some studies have suggested that doses markedly lower than our current dose could be used. A recent study by Hatter et al. [20] showed that lower dose, 50 µg of intrathecal morphine, was not inferior to 100 µg. Thus, it could be speculated that if in previously healthy parturients lower doses could be used, even better bladder function would be seen.

A significantly faster mobilization after caesarean section is clinically a very important finding, since immobilization is a well-known risk factor for venous thromboembolism (VTE) [21]; one of the leading causes of maternal death in the developed countries [22]. A prior cohort study has indicated that the rate of VTE during the third trimester was six times higher compared to the time outside pregnancy and, moreover, during the first six weeks postpartum there was a 22-fold increase in the risk of VTE [23]. Research has shown that the incidence of VTE after caesarean section among low-risk patients is 2 per 1 000 caesarean Sect. [24]. Previous research has also established that early mobilization reduces postoperative pain [25] and elevates peripheral oxygen saturation as well as arterial oxygen pressure [26].

Our results indicate no difference in patient satisfaction between the groups since both groups were highly satisfied, reporting median satisfaction VAS scores between 90 and 95. The results of this study show no difference in the length of hospital stay. In both groups, most patients were discharged during the second postoperative day. National Institute for Health and Care Excellence (NICE) guideline suggest discharge after 24 h for women who are recovering well, do not have fever, or complications after caesarean Sect. [27] and this can be implemented in our units as well. However, in Finland pediatricians examine all newborns before discharge, usually at the age of 24–48 h [28]. Therefore, the women’s enhanced recovery after a caesarean section is not expected to significantly shorten the length of stay.

This study found that the median time to the first voiding after the end of the caesarean section without an indwelling urinary catheter was 7.6 h. This finding is consistent with previous studies [29]. In this study, 47% of these women needed catheterization in the maternity ward at least once due to urinal retention or feeling unwell. The level of urinary retention was found to be higher than the levels reported in previous studies [29, 30], possibly due to differences in definition and protocols for re-catheterization. It is also worth mentioning that performing a cesarean without a urinary catheter requires bladder scanning and monitoring in the maternity ward and thus increases the workload. Prior studies have noted that limited resources may lead staff to pursue convenience and thereby suboptimize the regular postoperative follow-up and treatment [31, 32]. These factors may, to some extent, have influenced the decision to catheterize the patients. While earlier mobilization is beneficial for the parturient and ultimately enables more independent self-care, it also requires careful monitoring and treatment from the staff.

In summary, our findings suggest that a catheter-free perioperative protocol for uncomplicated caesarean section is associated with earlier postoperative mobilization without compromising patient satisfaction or operative safety. In the multivariable analysis, the absence of perioperative urinary catheterization was the strongest factor associated with shorter time to mobilization, supporting catheter-free management as an important and modifiable component of enhanced recovery pathways. Diagnoses of breech presentation and fear of childbirth were also independently associated with earlier mobilization; however, these associations were not as strong. These indications are generally not associated with maternal conditions that impair postoperative recovery, which may partly explain these findings. In addition, women with fear of childbirth may receive more extensive preoperative counselling and support, which could potentially enhance adherence to postoperative mobilization recommendations. However, these explanations remain speculative. Fear of childbirth was significantly more common in group 2. As the study included consecutive patients, this difference is unlikely to reflect intentional selection and may instead be related to the increasing prevalence of fear of childbirth and a growing preference for cesarean delivery among affected women.

This study has several strengths that enhance the validity and generalizability of its findings. The study population represented a relatively homogeneous, low-risk cohort of patients undergoing cesarean delivery at a single hospital, where standardized clinical protocol and consistent documentation practices were used. The use of reliable patient-level data in a real-world clinical setting further strengthens the validity and practical relevance of the findings. To the best of our knowledge, this is the first study to evaluate the impact of a change in clinical catheterization practice on mobilization among patients undergoing cesarean delivery.

However, this study also has some limitations. Firstly, retrospective design is an inherent limitation, as data collected from patient records may be subject to information bias. Secondly, the recruitment period for group 2 began more than one year after that of the first group, by which time the protocol for cesarean delivery without an indwelling urinary catheter had been successfully adopted in our hospital. Differences in staff experience over time may therefore have introduced bias, although the institutional protocol for early mobilization remained similar during both study periods.

## Conclusion

The two groups of this study differ from each other with respect to the use or planned duration of urinary catheterization. The prior pilot study showed that the intent to limit the duration of urinary catheterization was associated with shorter time to mobilization. In the current study, we provide further evidence of the association between perioperative urinary catheterization and mobilization and highlight catheter management as a potentially modifiable factor in optimizing postoperative recovery. Without a urinary catheter, patients can move more freely without possible irritation or pain in the urinary tract. The need to void independently and walk to the bathroom might also motivate patients to mobilize earlier. These findings support the growing body of evidence advocating catheter-free approaches as a safe and beneficial practice in uncomplicated cases. However, careful monitoring of bladder function remains essential. This protocol aligns closely with the principles of ERAS^®^, underscoring the importance of patient-centered, evidence-based perioperative care and the enhancement of maternal care quality.

## Data Availability

The data that support the findings of this study are not openly available due to reasons of regulatory data sensitivity control and are available from the corresponding author upon reasonable request and pending institutional data regulatory authorization.

## Abbreviation

ERAS: Enhanced Recovery After Surgery
